# PGT-A versus expectant management for fertile patients with recurrent pregnancy loss and prior aneuploidy: a dual-center, real-world study of 5-year cumulative pregnancy outcomes

**DOI:** 10.3389/fendo.2026.1926944

**Published:** 2026-08-27

**Authors:** Chunyan Wu, Ruochun Lian, Suhua Zou, Yan Xu, Yong Zeng, Qiong Wang, Lu Luo

**Affiliations:** 1 Reproductive Medicine Center, Department of Obstetrics and Gynecology, The First Affiliated Hospital, Sun Yat-sen University, Guangzhou, China; 2 Department of Obstetrics and Gynaecology, Li Ka Shing Faculty of Medicine, The University of Hong Kong, Hong Kong, Hong Kong SAR, China; 3 Shenzhen Key Laboratory of Reproductive Immunology for Peri-implantation, Shenzhen Zhongshan Institute for Reproductive Medicine and Genetics, Shenzhen Zhongshan Obstetrics and Gynecology Hospital, Shenzhen, China

**Keywords:** cost-effectiveness analysis, embryonic aneuploidy, expectant management, preimplantation genetic testing for aneuploidy, recurrent pregnancy loss

## Abstract

**Introduction:**

This study evaluated the effectiveness and cost-effectiveness of preimplantation genetic testing for aneuploidy (PGT-A) versus expectant management (EM) for achieving live birth among fertile patients with recurrent pregnancy loss (RPL) and prior aneuploid pregnancy loss.

**Methods:**

This dual-center retrospective cohort study included 216 fertile patients with RPL and at least one previous aneuploid pregnancy loss. Seventy-one patients chose EM and 145 chose PGT-A. Propensity score matching (PSM), multivariable logistic regression, and subgroup analyses were performed. The primary outcome was conception leading to live birth (CLLB) within 12 months. Secondary outcomes included CLLB at 24 and 36 months, cumulative live birth (CLB) at 5 years, time to pregnancy leading to live birth (TPLB), early miscarriage per clinical pregnancy, and incremental cost-effectiveness ratios (ICERs).

**Results:**

After PSM, 71 matched pairs were analyzed. CLLB within 12 months was lower in the PGT-A group than in the EM group (52.1% vs. 81.7%; adjusted odds ratio 0.27, 95% confidence interval 0.13–0.53; P < 0.001). Five-year CLB rates were similar (94.4% vs. 87.3%; P = 0.15), whereas TPLB was longer with PGT-A (median 8.6 vs. 3.0 months; P < 0.001). Early miscarriage rates per clinical pregnancy did not differ significantly (7.4% vs. 12.4%; adjusted odds ratio 0.42; P = 0.09). The ICERs per additional live birth and per miscarriage prevented were ¥1,203,800 and ¥1,504,800, respectively.

**Discussion:**

In fertile patients with RPL and prior aneuploidy, PGT-A and EM were associated with similar 5-year cumulative live birth rates, whereas EM was associated with a higher probability of CLLB within 12 months and a shorter TPLB. A possible reduction in miscarriage with PGT-A remains uncertain and warrants further investigation.

## Introduction

The clinical utility of preimplantation genetic testing for aneuploidy (PGT-A) remains debated (1, 2). For the general infertile population, PGT-A does not significantly improve cumulative live birth rates (CLBR) (1), particularly in non-advanced-age women (3). This is reflected in consensus recommendations from the European Society of Human Reproduction and Embryology (ESHRE) and the American Society for Reproductive Medicine (ASRM), both advising against its routine use in unselected populations (2, 4).

In contrast, as embryonic aneuploidy is a major cause of miscarriage (5), recurrent pregnancy loss (RPL) is widely regarded as a legitimate indication for PGT-A (2, 6). However, evidence of its efficacy in RPL is inconsistent. Several retrospective studies reported improved per-patient live birth rates and lower miscarriage rates with PGT-A compared with conventional IVF (7, 8), whereas a prospective cohort study found no significant differences (9), and an analysis of the Society for Assisted Reproductive Technology (SART) database suggested improved live birth rates per transfer without significantly reducing miscarriage rates (10). These conflicting findings likely reflect differences across study populations in the proportion of miscarriages attributable to embryonic aneuploidy.

Critically, PGT-A cannot benefit patients whose losses are due to euploid causes; therefore, its potential value lies primarily in those at high risk of embryonic aneuploidy. Patients with RPL and a confirmed prior aneuploid miscarriage represent a subgroup considered most likely to benefit. Limited evidence shows that in women under 38 years, unexplained RPL patients have higher embryonic aneuploidy rates than age-matched controls (11), and the number of prior aneuploid losses may independently predict aneuploidy in biopsied embryos (12). Consequently, many scholars have identified RPL with prior aneuploid loss as a key population for focused PGT-A research (13).

Despite this theoretical rationale, robust evidence evaluating PGT-A specifically in this population remains scarce. Moreover, a large proportion of RPL patients are fertile, so the relevant question is not whether PGT-A improves IVF outcomes but whether it outperforms natural conception. To date, no study has directly compared the PGT-A pathway versus expectant management (EM) in fertile RPL patients with prior aneuploidy—a pivotal question that also lacks cost-effectiveness data.

To address this gap, this pilot study investigates, in fertile RPL patients with prior aneuploidy, whether PGT-A or EM leads to differences in conception leading to live birth within 12 months (primary endpoint), 5-year CLBR, and time to live birth, while also assessing cost-effectiveness, thereby providing evidence on clinical and economic outcomes to inform decision-making.

## Materials and methods

### Study design and participants

This was a retrospective cohort study using real-world data from two reproductive centers in China: The First Affiliated Hospital of Sun Yat-sen University and Zhongshan Obstetrics and Gynecology Hospital. We included fertile women with RPL and prior aneuploidy. Inclusion criteria were age 25–40 years, at least two pregnancy losses with at least one confirmed aneuploidy documented by chromosomal analysis of products of conception (POC), and no prior assisted reproductive technology (ART) cycles. Exclusion criteria were known chromosomal abnormalities, untreated RPL of known etiology, and a history of infertility or any factor known to impair fertility. Patients opted either for PGT-A utilizing the next-generation sequencing (NGS) platform or for EM without medical intervention. The study was approved by the institutional review boards of the participating centers [approval No. 2020(081)]. Informed consent was waived due to the retrospective nature of the study.

### Study interventions

All couples received genetic counseling before interventions. For those undergoing PGT-A, ovarian stimulation used gonadotropin-releasing hormone (GnRH) agonist or antagonist protocols as previously described (11, 14). When three or more follicles reached ≥18 mm, oocyte maturation was triggered with triptorelin (0.2 mg; Decapeptyl, Ferring Pharmaceuticals, Germany) alone, or combined with 2,000 IU–6,000 IU human chorionic gonadotropin (hCG; Lizhu Pharmaceutical Trading Co., China), or 4,000 IU–10,000 IU hCG alone (Pregnyl, Merck).

Oocyte retrieval was performed 34 h–36 h later. Intracytoplasmic sperm injection (ICSI) was used for fertilization. Normal fertilization was confirmed 16 h–18 h later by two pronuclei and two polar bodies. Embryo culture and blastocyst biopsy followed published protocols (15). Genetic testing used an NGS-based assay (16). Blastocysts biopsied for PGT-A were limited to day 5 and day 6 embryos; day 7 blastocysts were not biopsied. Briefly, six to eight trophectoderm cells were lysed by laser; whole-genome amplification used multiple displacement amplification (Qiagen REPLI-g kits). Cytogenetic analysis used the MiSeq system (Illumina) and BlueFuse Multi software. Embryos diagnosed as mosaic were not transferred in accordance with our center’s clinical protocol at the time of the study. Endometrial preparation and embryo transfer were performed as described (11, 15, 16).

Patients choosing EM were encouraged to attempt natural conception without medical intervention. If a couple did not conceive within 12 months, a comprehensive infertility evaluation was performed.

### Follow-up and data collection

Follow-up began at the start of the first menstrual cycle for natural conception or at the onset of ovarian stimulation for PGT-A. It ended at the first live birth or after a maximum of 5 years from the initial conception attempt, provided that at least one complete PGT-A cycle was finished and all viable embryos had been transferred.

Conception was defined as hCG-positive. Clinical pregnancy was defined as the presence of a gestational sac at 6–7 weeks of gestation. Biochemical loss was pregnancy loss before the appearance of a gestational sac. Early clinical miscarriage was pregnancy loss before 12 weeks with a gestational sac. Live birth was delivery of a neonate after 24 weeks.

### Outcome measures

The primary outcome was conception leading to live birth (CLLB) within 12 months, defined as a conception occurring within 12 months from treatment start that subsequently resulted in a live birth. Secondary outcomes were CLLB at 24 and 36 months, cumulative live birth (CLB) rate at 5 years, time to pregnancy leading to live birth (TPLB), early clinical miscarriage per clinical pregnancy, and gestational age at live birth. In the ITT analysis, CLLB outcomes were assessed on a per-patient basis, counting each patient’s first live birth, regardless of the number of embryo transfer attempts or conception attempts during the 5-year follow-up period.

Economic outcomes, including direct medical costs and incremental cost-effectiveness ratios (ICERs), were also evaluated.

### Statistical analyses

Baseline characteristics were compared using univariate tests. Propensity score matching (PSM) was used to reduce selection bias: 71 matched pairs were created using a caliper of 0.1 based on age, body mass index (BMI), parity, number of prior clinical miscarriages, and number of prior aneuploid miscarriages (17). Covariate balance was assessed using standardized mean differences (SMDs), with values ≤0.1 indicating good balance. After matching, all covariates were well balanced (all SMDs ≤0.1). Multivariable logistic regression and age-stratified analyses were performed to adjust for confounders and explore effect modification.

Missing data: one patient in the EM group was lost to follow-up after achieving pregnancy; this patient was included in the ITT analysis (counted as treatment failure) and excluded from the per-protocol analysis. No missing data were present for any baseline covariates or outcome variables.

All analyses used IBM SPSS Statistics (version 25.0; IBM Corp., USA) and EasyR (https://www.easyr.cc). Normally distributed continuous variables were compared with Student’s t-test, non-normal with Mann–Whitney *U* test, and categorical variables with chi-squared test. A two-sided P <0.05 was considered significant.

### Cost-effectiveness analysis

A cost-effectiveness analysis was performed on the matched cohort (71 pairs) from a healthcare perspective. Direct medical costs (in Chinese yuan, ¥) included medications, procedures, laboratory tests, and hospital visits. Mean costs per patient were estimated with 95% confidence intervals (CI). The primary effectiveness measure was live birth; the secondary measure was early miscarriage prevented. The incremental cost-effectiveness ratio (ICER) was calculated as the difference in mean cost between the PGT-A and EM groups divided by the difference in effectiveness between the two groups. Uncertainty was assessed using bootstrap resampling (1,000 replicates) to generate 95% CIs for ICERs and cost-effectiveness acceptability curves. In the base-case analysis, costs were not discounted (0%). In sensitivity analyses, annual discount rates of 5% and 8% were applied by assigning each patient’s total cost to the midpoint between study entry and the first live birth or the end of follow-up, whichever occurred first.

Given the absence of an established WTP threshold for an additional live birth in reproductive medicine, the conventional QALY-based benchmark of one to three times annual GDP per capita was used as a broad reference. A hypothetical WTP threshold of ¥6 million per additional live birth was selected for the scenario analysis based on China’s 2024 GDP per capita and the study context. This value was used for exploratory purposes and should not be interpreted as a guideline-endorsed threshold (18, 19).

## Results

### Demographic and clinical characteristics of study participants

**Figure 1:**
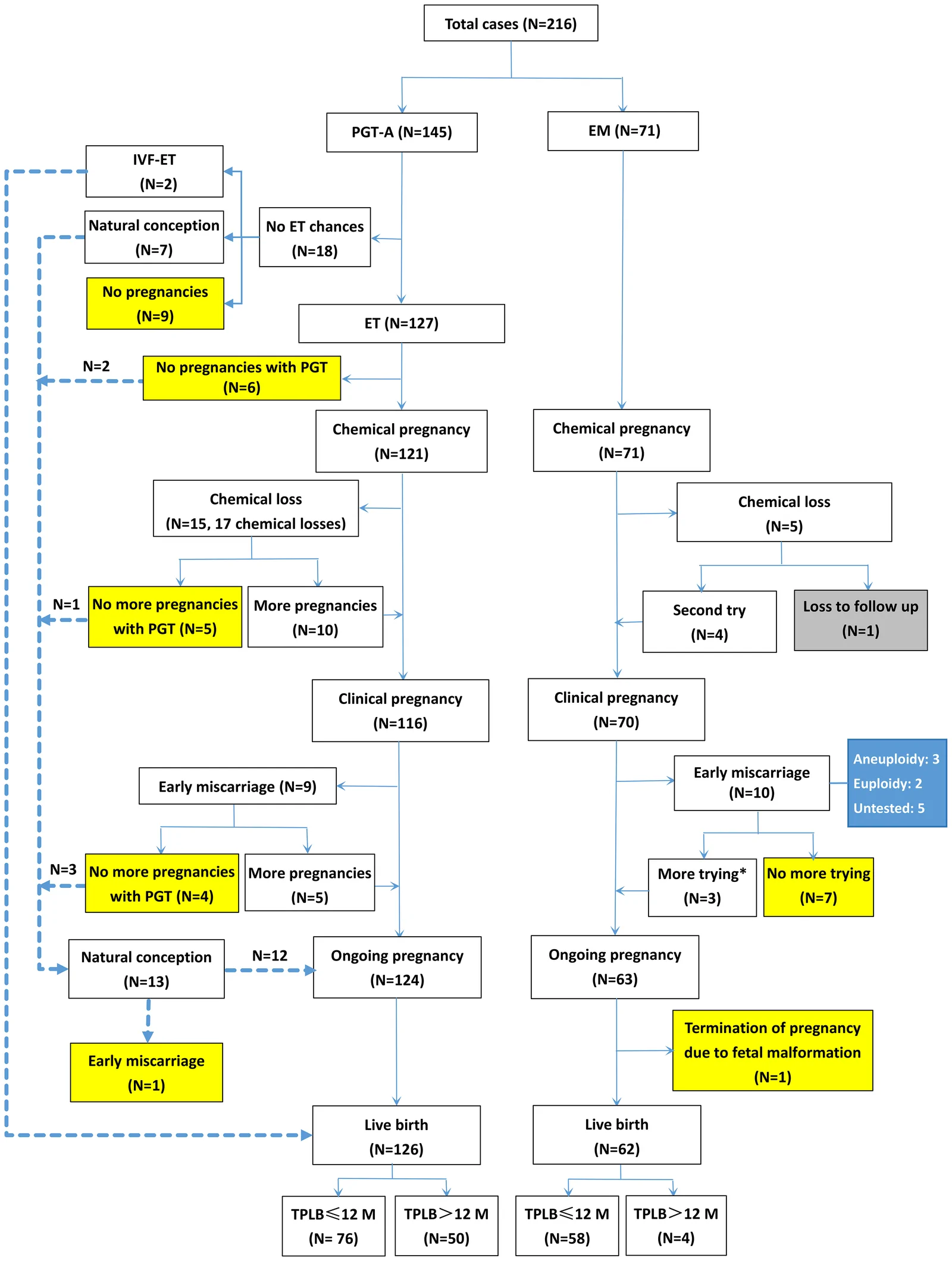
Flow chart of patient inclusion, matching, and follow-up. PGT-A, preimplantation genetic testing for aneuploidy; EM, expectant management; ITT, intention-to-treat; PSM, propensity score matching.

The flow chart (**Figure 1**) summarizes patient outcomes. One patient in the EM group was lost to follow-up after conception. Two patients (2.8%) in the EM group crossed over to PGT-A after failing to conceive or experiencing miscarriage during expectant management and achieved live birth; 17 patients (11.7%) in the PGT-A group who did not achieve live birth through PGT-A eventually achieved live birth through non-PGT-A pathways (15 natural conceptions and two conventional IVF cycles). All patients remained in their original treatment groups for the ITT analysis. **Table 1** presents baseline characteristics per ITT before and after PSM, along with SMDs for each covariate. After matching, all variables were comparable between groups, with all SMDs ≤0.1, indicating good balance.

**Table 1:** Baseline characteristics before and after propensity score matching (intention-to-treat population).

Characteristic	Before matching				After matching			
	PGT-A	EM	*P*	*SMD*	PGT-A	EM	*P*	*SMD*
N	145	71			71	71		
Female age, y	33 (31, 36)	33 (31, 35)	0.44	0.20	33 (31, 36)	33 (31, 35)	0.30	0.07
Male age, y	34 (31, 37)	33 (32, 36)	0.54	0.22	34 (32, 37)	34 (32, 36)	0.79	0.10
BMI, kg/m^2^	21.0 (20.0, 23.1)	21.2 (19.7, 22.4)	0.93	0.08	21.0 (20.0, 22.3)	21.2(19.7, 22.4)	0.99	0.04
Basal FSH, IU/L	5.9 (4.8, 6.8)	5.6 (4.9, 7.2)	0.88	0.15	5.7 (4.7, 6.8)	5.6 (4.9, 7.2)	0.83	0.11
Parity	23 (15.9%)	17 (23.9%)	0.15	0.27	13 (18.3%)	17 (23.9%)	0.41	0.06
The number of prior miscarriages	3 (2, 3)	2 (2, 3)			2 (2, 3)	2 (2, 3)		
2	69 (47.6%)	42 (59.1%)	0.14	0.32	36 (50.7%)	42 (59.1%)	0.49	0.09
3	48 (33.1%)	22 (31.0%)			24 (33.8%)	22 (31.0%)		
≥4	28 (19.3%)	7 (9.9%)			11 (15.5%)	7 (9.9%)		
The number of prior aneuploid miscarriages	1 (1, 1)	1 (1, 1)			1 (1, 1)	1 (1, 1)		
1	128 (88.3%)	65 (91.5%)	0.46	0.11	66 (93.0%)	65 (91.5%)	0.75	0.05
2	17 (11.7%)	6 (8.5%)			5 (7.0%)	6 (8.5%)		

BMI, body mass index; FSH, follicle-stimulating hormone.

### PGT-A cycle and embryo biopsy outcomes

A total of 968 blastocysts were biopsied and analyzed by next-generation sequencing. The euploidy rate was 53.3% (516/968); 290 (30.0%) were aneuploid, 81 (8.4%) segmentally abnormal, 67 (6.9%) were mosaic, and 14 (1.4%) were inconclusive. Thirteen patients (9.0%) had no euploid embryo available after biopsy. A total of 191 frozen-thawed single embryo transfers were performed in the PGT-A group, with a mean of 1.3 transfers per patient (range 0–4). Detailed cycle and embryo biopsy outcomes are provided in **Supplementary Table 1**.

### Overall cumulative live birth outcomes

**Figure 2:**
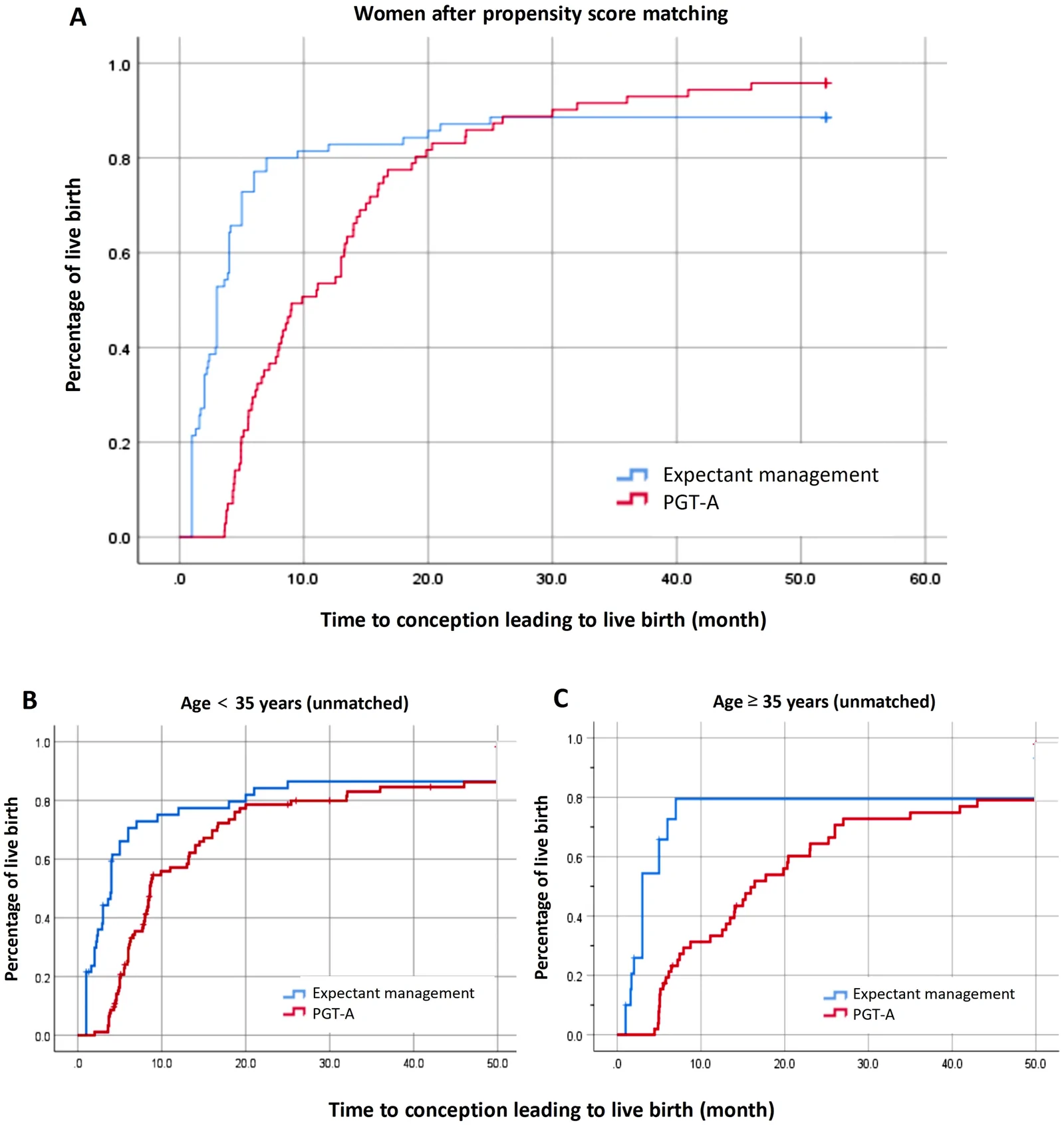
Time to pregnancy leading to live birth (TPLB)—Kaplan–Meier curves. **(A)** Overall comparison between the PGT-A and EM groups. **(B)** Subgroup of women aged <35 years. **(C)** Subgroup of women aged ≥35 years. Median TPLB (months) and log-rank P values are shown. PGT-A, preimplantation genetic testing for aneuploidy; EM, expectant management.

ITT analysis of pregnancy outcomes before and after PSM is shown in **Table 2**. After matching, the primary outcome, CLLB within 12 months, was significantly lower in the PGT-A group than in the EM group (52.1% vs. 81.7%; absolute difference −29.6%; P <0.001). CLLB at 24 months (84.5% vs. 84.5%; P = 1.0) and 36 months (91.5% vs. 87.3%; P = 0.41) were comparable. The 5-year cumulative live birth (CLB) rate did not differ (94.4% vs. 87.3%; P = 0.15). Time to pregnancy leading to live birth (TPLB) was longer with PGT-A (median 8.6 vs. 3.0 months; P <0.001; **Figure 2A**). Median gestational age at live birth was shorter in the PGT-A group (38 vs. 39 weeks; P = 0.01).

**Table 2:** Clinical outcome comparisons (per intention-to-treat).

Outcome	Before matching			After matching		
	PGT-A	EM	*P*	PGT-A	EM	*P*
N	145	71		71	71	
CLLB within 12 months	76 (52.4%)	58 (81.7%)	<0.001	37 (52.1%)	58 (81.7%)	<0.001
CLLB within 24 months	109 (75.2%)	60 (84.5%)	0.12	60 (84.5%)	60 (84.5%)	1.0
CLLB within 36 months	121 (83.4%)	62 (87.3%)	0.46	65 (91.5%)	62 (87.3%)	0.41
CLB at 5 years	126 (86.9%)	62 (87.3%)	0.93	67 (94.4%)	62 (87.3%)	0.15
TPLB, months	8.3 (5.5, 16.0)	3.0 (1.2, 5.0)	<0.001	8.6 (5.2, 14.5)	3.0 (1.2, 5.0)	<0.001
Gestational age at birth, weeks	38 (38, 39)	39 (38, 40)	0.001	38 (38, 39)	39 (38, 40)	0.01

CLLB, conception leading to live birth; CLB, cumulative live birth; TPLB, time to pregnancy leading to live birth.

### Age-stratified cumulative outcomes

Age-stratified analyses are presented in **Supplementary Table 2**. Consistent with the whole cohort, CLLB at 12 months was lower with PGT-A in both age subgroups, while 5-year CLB was similar. TPLB was longer with PGT-A in both younger and older patients (**Figures 2B, C**), and the difference was more pronounced in women aged ≥35 years (<35 years: 8.0 vs. 3.0 months; ≥35 years: 11.1 vs. 3.0 months; both P <0.001).

### Multivariable and effect modification analyses for CLLB within 12 months

Multivariable logistic regression adjusting for age, BMI, parity, number of prior early miscarriages, number of prior aneuploid miscarriages, and basal FSH confirmed that PGT-A remained independently associated with a lower probability of CLLB at 12 months (aOR 0.27, 95% CI 0.13–0.53; P <0.001).

Subgroup analyses (**Supplementary Figure 1**) showed a consistent direction of effect (all aORs <1), though the magnitude varied. The number of prior miscarriages significantly modified the effect (P for interaction = 0.047), with a larger reduction in CLLB among patients with two prior miscarriages than in those with ≥3. A borderline interaction was observed for ovarian reserve (FSH ≥7 vs. <7 IU/L, the 75th percentile; P = 0.073), suggesting a stronger negative association in women with decreased reserve.

### Clinical miscarriage risk analyses

**Supplementary Table 3** shows baseline characteristics of the per-clinical-pregnancy cohort (based on actual conception mode); **Supplementary Table 4** summarizes miscarriage outcomes. In the primary per-clinical-pregnancy analysis, the unadjusted early miscarriage rate did not differ significantly between groups (7.4% vs. 12.4%; P = 0.09). Age-stratified analysis showed no significant difference (<35 years: 7.6% vs. 13.4%, P = 0.06; ≥35 years: 7.0% vs. 9.1%, P = 0.79). After multivariable adjustment, PGT-A was not independently associated with miscarriage risk (aOR 0.42, 95% CI 0.15–1.14; P = 0.09). A higher number of previous early miscarriages was a strong independent risk factor (aOR 3.74, 95% CI 1.28–10.97; P = 0.02). In the exploratory per-patient ITT analysis (full cohort), the cumulative miscarriage rate was 6.9% in the PGT-A group versus 14.1% in the EM group (P = 0.037). A significant reduction was observed in women under 35 years (6.5% vs. 17.6%; P = 0.006), but not in those ≥35 years (7.8% vs. 5.3%; P = 0.60).

### Cost-effectiveness analysis

Cost-effectiveness analysis was performed on the matched cohort (**Supplementary Table 6**). Mean direct medical costs per patient were ¥92,300 (95% CI ¥84,000–¥101,200) in the PGT-A group and ¥1,100 (95% CI ¥800–¥1,400) in the EM group. The incremental cost-effectiveness ratio (ICER) per additional live birth was ¥1,203,800; the ICER per miscarriage prevented was ¥1,504,800. Bootstrap resampling (1,000 replicates) gave mean ICERs of ¥1,229,400 (95% CI −¥5,794,100 to ¥6,245,100) for live birth and ¥1,167,600 (95% CI −¥5,994,600 to ¥6,225,100) for miscarriage prevention.

The cost-effectiveness plane and cost-effectiveness acceptability curve for additional live birth, based on the hypothetical WTP threshold specified in the **Materials and methods**, are presented in **Supplementary Figures 2**, **3**, respectively.

The results were generally robust to alternative discounting assumptions. Applying annual discount rates of 5% and 8% resulted in only modest reductions in the estimated ICERs compared with the undiscounted analysis, without materially changing the overall findings (**Supplementary Table 7**).

## Discussion

### Main findings

This study provides the first direct comparison between PGT-A and EM in fertile RPL patients with a history of aneuploidy, with a primary focus on the 12-month CLLB. Our real-world, dual-center data show that PGT-A significantly reduces the probability of live birth within 12 months compared with EM. Despite both management strategies yielding similarly high cumulative live birth rates by 5 years, patients in the EM group achieved live birth more rapidly. Together with the cost-effectiveness analysis, these findings indicate that PGT-A offers no clear clinical or cost-saving advantage over EM in this population.

### Strengths and limitations

This study has several strengths. First, to our knowledge, no previous study has directly compared comprehensive PGT-A with EM in this specific patient population—a clinically important but previously overlooked subgroup. Most existing research compared PGT-A with IVF in infertile populations (7–10), which does not reflect the clinical reality for these patients. Our study therefore addresses the real-world question that truly matters to them. Second, we used a comprehensive set of outcome measures covering effectiveness, efficiency, and cost-effectiveness, providing more complete information for comparing this complex treatment pathway. Most previous studies relied on a single endpoint, which has notable limitations. For example, per-transfer outcomes (20–24) exclude poor-prognosis patients without transferable embryos; miscarriage rate per pregnancy (21, 22) fails to capture the even larger number of non-pregnant cases; and cumulative live birth rate alone does not capture treatment efficiency (1, 2). In contrast, our multidimensional approach simultaneously assesses these domains based on ITT and per-pregnancy analyses using actual conception mode. This real-world approach provides a holistic view of the value of PGT-A, which is essential for clinical decision-making around a complex and expensive add-on. Third, we used a long follow-up period up to 5 years and applied rigorous statistical methods, including propensity score matching, multivariable regression, and subgroup analyses, to minimize confounding and explore effect modification.

Limitations should be acknowledged. One limitation is the retrospective design, which may have introduced selection bias. For example, PGT-A patients had more consistent follow-up than EM patients, who may have missed more visits. Treatment allocation was based on patient choice rather than randomization, and unmeasured factors such as socioeconomic status, psychological stress, and treatment preference may have influenced both treatment selection and reproductive outcomes. Although we applied PSM and multivariable adjustment to balance measured confounders, residual confounding cannot be excluded.

Another limitation is the modest sample size, particularly in subgroups. For the primary endpoint, the sample size was sufficient, but for secondary endpoints like miscarriage (a rare event), statistical power may have been insufficient; the wide confidence intervals for ICERs reflect this limitation. Furthermore, among the 10 miscarriages that occurred in the EM group during follow-up, only five underwent POC testing, limiting estimation of recurrent aneuploidy risk. This remains an important clinical question that warrants dedicated investigation in future studies. The cost analysis also did not include indirect or long-term pediatric costs, so the true economic burden may be underestimated.

Finally, our study focused specifically on fertile RPL patients with documented aneuploid miscarriage from two reproductive centers. Therefore, the findings should not be extrapolated to infertile patients, women without documented embryonic aneuploidy, patients with other RPL etiologies, or populations in different healthcare systems. The cost-effectiveness estimates are also context-specific and may not be generalizable to other settings.

### Interpretation

Why did EM achieve a higher CLLB rate within 12 months? Fertile RPL patients have a good natural prognosis. Published data show that the CLBR after natural conception in RPL with prior aneuploid loss reaches 70%–80% (25, 26), with a median time to pregnancy of about 2–3 months (27), consistent with our EM group. In contrast, the reported cumulative live birth rate after one PGT-A retrieval cycle is highly dependent on patient characteristics such as age and ovarian reserve, with an overall rate of approximately 50% (7) in RPL populations. The PGT-A pathway involves multiple steps, including ovarian stimulation, retrieval, biopsy, genetic testing, and frozen-embryo transfer, taking at least 3–4 months even under ideal conditions. In reality, a proportion of women, especially those with diminished ovarian reserve, fail to obtain a transferable euploid embryo and require repeated stimulation cycles. Moreover, the high cost and psychological burden often lead to treatment hesitancy, cycle cancellations, and longer intervals after a failed cycle. Consequently, within a 12-month window, most patients can complete only one PGT-A retrieval cycle, and only a few achieve two. Notably, the expectation that PGT-A might shorten time to live birth has been largely derived from studies comparing PGT-A with IVF in populations that often include infertile patients (28, 29)—a different clinical context from our study population. Our findings suggest that this time-saving benefit may not generalize to fertile RPL patients when compared with natural conception. By contrast, natural conception is simple, less stressful, and can be attempted repeatedly, markedly increasing the cumulative chance of success within the first year. In summary, the combination of higher natural fertility, fewer procedural barriers, and the ability for repeated attempts explains why EM achieved a higher CLLB rate within the first year.

Regarding miscarriage, a consistent trend toward lower rates with PGT-A was observed across analyses, but the differences did not reach statistical significance in the pre-specified primary per-clinical-pregnancy analysis. This may partly reflect insufficient statistical power to detect a modest difference in a relatively infrequent event, but importantly, even if a true effect exists, the absolute risk reduction appeared limited. The overall picture is further complicated by several patient-specific factors. In particular, the number of prior miscarriages emerged as a strong independent risk factor (aOR 3.74; 95% CI 1.28–10.97; p = 0.02), and the risk of recurrent aneuploidy—which may vary by maternal age and other patient characteristics—further complicates the picture. The fact that the exploratory benefit of PGT-A in miscarriage reduction was observed only in women under 35 years, and was not confirmed in the primary per-clinical-pregnancy analysis or multivariable adjustment, suggests that these complex risk factors may partially influence or confound the observed associations. This underscores the need for future studies to better disentangle the interplay between patient history, biological predisposition, and treatment effects.

The age-dependent pattern—a possible benefit in women under 35 years but not in older women—may be explained by the unique nature of our study population. Younger women with recurrent aneuploid loss could have an intrinsic predisposition (e.g., genetic variants affecting meiosis) (30–32), whereas aneuploid miscarriages in older women are likely random age-related events, and their diminished ovarian reserve or reduced response often dilutes any potential benefit of PGT-A (33). This finding, although exploratory, suggests that future research should focus on a composite biomarker set—integrating age, prior miscarriage history, and novel intrinsic biomarkers—to define a subset of younger patients who might truly benefit.

The shorter median gestational age in the PGT-A group, although statistically significant, is of uncertain clinical relevance. It may reflect subtle biological differences between pregnancies conceived after frozen-thawed embryo transfer and those conceived naturally, or a higher rate of elective cesarean section in the PGT-A group, potentially related to pregnancy complications or patient/physician preference for earlier elective delivery for safety considerations. However, given the retrospective design, we were unable to further explore these contributing factors.

Overall, our findings do not simply declare one strategy as superior to the other based on a single measurement; instead, they provide a comprehensive view of the trade-offs between effectiveness (34), efficiency, and cost. This information can help clinicians and patients make individualized decisions based on personal priorities—for example, whether speed to live birth, avoidance of miscarriage, or cost minimization matters most.

## Conclusion

In fertile RPL patients with prior aneuploidy, PGT-A and EM yield similar 5-year cumulative live birth rates, though EM achieves CLLB more efficiently within 12 months. PGT-A might offer some benefit in miscarriage reduction; however, the risk of subsequent miscarriage appears to be influenced by a more complex set of factors that merit further investigation. Patients should be fully counseled about these trade-offs when making treatment decisions.

## Data Availability

The raw data supporting the conclusions of this article will be made available by the authors, without undue reservation.

## Supplementary material

Supplementary Figure 1 Forest plot of subgroup analyses for CLLB within 12 months. Adjusted odds ratios (aOR) with 95% confidence intervals (CI) are shown for each subgroup. The model was adjusted for age, BMI, parity, number of prior early miscarriages, number of prior aneuploid miscarriages, and basal FSH. P values for interaction are presented on the right. Sample sizes for each subgroup are indicated in parentheses. PGT-A: preimplantation genetic testing for aneuploidy; EM: expectant management; FSH: follicle-stimulating hormone.

Supplementary Figure 2 Cost-effectiveness plane for each additional live birth (1000 bootstrap replicates). The x-axis shows incremental live births (n); the y-axis shows incremental cost (10,000 Yuan). The diagonal line is the willingness-to-pay (WTP) threshold (¥6 million per live birth). Base-case ICER = ¥1.20 million; bootstrap mean = ¥1.23 million.

Supplementary Figure 3 Cost-effectiveness acceptability curve for each additional live birth. The x-axis is the WTP threshold (10,000 Yuan per live birth); the y-axis is the probability that PGT-A is cost-effective. The vertical dashed line marks the ¥6 million threshold.

## References

[1] Cornelisse S Zagers M Kostova E Fleischer K van Wely M Mastenbroek S . Preimplantation genetic testing for aneuploidies (abnormal number of chromosomes) in *in vitro* fertilization. Cochrane Database Syst Rev. (2020) 9:CD5291. doi: 10.1002/14651858.CD005291.pub3 32898291 PMC8094272

[2] Lundin K Bentzen J Bozdag G Ebner T Harper J ESHRE Add-ons working group . Good practice recommendations on add-ons in reproductive medicine. Hum Reprod. (2023) 38:2062–104. doi: 10.1093/humrep/dead184 37747409 PMC10628516

[3] Yan J Qin Y Zhao H Sun Y Gong F Li R . Live birth with or without preimplantation genetic testing for aneuploidy. N Engl J Med. (2021) 385:2047–58. doi: 10.1056/NEJMoa2103613 34818479

[4] Practice Committees of the American Society for Reproductive Medicine . The use of preimplantation genetic testing for aneuploidy: A committee opinion. Fertil Steril. (2024) 122:421–34. doi: 10.1016/j.fertnstert.2024.05.166 38762806

[5] Dimitriadis E Menkhorst E Saito S Kutteh W Brosens J . Recurrent pregnancy loss. Nat Rev Dis Primers. (2020) 6:98. doi: 10.1038/s41572-020-00228-z 33303732

[6] Morales C . Current applications and controversies in preimplantation genetic testing for aneuploidies (PGT-a) in *in vitro* fertilization. Reprod Sci. (2024) 31:66–80. doi: 10.1007/s43032-023-01301-0 37515717

[7] Mei Y Lin Y Chen Y Zheng J Ke X Liang X . Preimplantation genetic testing for aneuploidy optimizes reproductive outcomes in recurrent reproductive failure: A systematic review. Front Med. (2024) 11:1233962. doi: 10.3389/fmed.2024.1233962 38384413 PMC10879326

[8] Pantou A Mitrakos A Kokkali G Petroutsou K Tounta G Lazaros L . The impact of preimplantation genetic testing for aneuploidies (PGT-a) on clinical outcomes in high risk patients. J Assist Reprod Genet. (2022) 39:1341–9. doi: 10.1007/s10815-022-02461-9 35338417 PMC9174385

[9] Sato T Sugiura-Ogasawara M Ozawa F Yamamoto T Kato T Kurahashi H . Preimplantation genetic testing for aneuploidy: A comparison of live birth rates in patients with recurrent pregnancy loss due to embryonic aneuploidy or recurrent implantation failure. Hum Reprod. (2019) 34:2340–8. doi: 10.1093/humrep/dez229 31811307

[10] Bhatt SJ Marchetto NM Roy J Morelli SS McGovern PG . Pregnancy outcomes following *in vitro* fertilization frozen embryo transfer (IVF-FET) with or without preimplantation genetic testing for aneuploidy (PGT-a) in women with recurrent pregnancy loss (RPL): A SART-CORS study. Hum Reprod. (2021) 36:2339–44. doi: 10.1093/humrep/deab117 34027546

[11] Liu X Fan Q Wang J Li R Xu Y Guo J . Higher chromosomal abnormality rate in blastocysts from young patients with idiopathic recurrent pregnancy loss. Fertil Steril. (2020) 113:853–64. doi: 10.1016/j.fertnstert.2019.11.016 32228881

[12] Zhang L Yang Y Wang W Luo L Zhang Z Wu J . Predicting risk of blastocyst aneuploidy among women with previous aneuploid pregnancy loss: A multicenter-data-based multivariable model. Hum Reprod. (2023) 38:2382–90. doi: 10.1093/humrep/dead202 37801294

[13] Papas R Kutteh W . A new algorithm for the evaluation of recurrent pregnancy loss redefining unexplained miscarriage: Review of current guidelines. Curr Opin Obstet Gynecol. (2020) 32:371–9. doi: 10.1097/GCO.0000000000000647 32590384

[14] Wang Q Luo L Lei Q Lin M Huang X Chen M . Low aneuploidy rate in early pregnancy loss abortuses from patients with polycystic ovary syndrome. Reprod BioMed Online. (2016) 33:85–92. doi: 10.1016/j.rbmo.2016.04.006 27157933

[15] Luo L Gu F Jie H Ding C Zhao Q Wang Q . Early miscarriage rate in lean polycystic ovary syndrome women after euploid embryo transfer - a matched-pair study. Reprod BioMed Online. (2017) 35:576–82. doi: 10.1016/j.rbmo.2017.07.010 28802704

[16] Tong J Niu Y Wan A Zhang T . Comparison of day 5 blastocyst with day 6 blastocyst: Evidence from NGS-based PGT-a results. J Assist Reprod Genet. (2022) 39:369–77. doi: 10.1007/s10815-022-02397-0 35013836 PMC8956767

[17] Luo L Fan X Jie H Gao Y Chen M Zhou C . Is it worth reducing twins to singletons after IVF-ET? A retrospective cohort study using propensity score matching. Acta Obstet Gynecol Scand. (2019) 98:1274–81. doi: 10.1111/aogs.13640 31081540

[18] Bertram MY Lauer JA De Joncheere K Edejer T Hutubessy R Kieny M-P . Cost–effectiveness thresholds: Pros and cons. Bull World Health Organ. (2016) 94:925–30. doi: 10.2471/BLT.15.164418 27994285 PMC5153921

[19] Cai D Shi S Jiang S Si L Wu J Jiang Y . Estimation of the cost-effective threshold of a quality-adjusted life year in China based on the value of statistical life. Eur J Health Econ. (2022) 23:607–15. doi: 10.1007/s10198-021-01384-z 34655364 PMC9135816

[20] Lee M Lofgren K Thomas A Lanes A Goldman R Ginsburg E . The cost-effectiveness of preimplantation genetic testing for aneuploidy in the United States: An analysis of cost and birth outcomes from 158,665 *in vitro* fertilization cycles. Am J Obstet Gynecol. (2021) 225:51–5. doi: 10.1016/j.ajog.2021.01.021 33539823

[21] Munne S Kaplan B Frattarelli J Child T Nakhuda G Shamma F . Preimplantation genetic testing for aneuploidy versus morphology as selection criteria for single frozen-thawed embryo transfer in good-prognosis patients: A multicenter randomized clinical trial. Fertil Steril. (2019) 112:1071–9. doi: 10.1016/j.fertnstert.2019.07.1346 31551155

[22] Murugappan G Shahine L Perfetto C Hickok L Lathi R . Intent to treat analysis of *in vitro* fertilization and preimplantation genetic screening versus expectant management in patients with recurrent pregnancy loss. Hum Reprod. (2016) 31:1668–74. doi: 10.1093/humrep/dew135 27278003

[23] Seckin S Forman EJ . Does PGT-a affect cumulative live birth rate? Curr Opin Obstetrics Gynecology. (2023) 35:216–23. doi: 10.1097/GCO.0000000000000865 37185353

[24] Simopoulou M Sfakianoudis K Maziotis E Tsioulou P Grigoriadis S Rapani A . PGT-a: Who and when? Alpha systematic review and network meta-analysis of RCTs. J Assist Reprod Genet. (2021) 38:1939–57. doi: 10.1007/s10815-021-02227-9 34036455 PMC8417193

[25] Yang Y Zhang X Zhang Y Ou M Wang C Lu Y . Chromosomal miscarriage and pregnancy outcomes in recurrent pregnancy loss. Reprod Fertil. (2025) 6:e250052. doi: 10.1530/RAF-25-0052 41283765 PMC12694013

[26] Li Y Zhou R Xia Z Meng L Huang M Hu P . Reproductive outcomes in couples with recurrent pregnancy loss after embryonic chromosomal microarray analysis. J Assist Reprod Genet. (2024) 41:161–70. doi: 10.1007/s10815-023-02971-0 37874532 PMC10789713

[27] Perfetto C Murugappan G Lathi R . Time to next pregnancy in spontaneous pregnancies versus treatment cycles in fertile patients with recurrent pregnancy loss. Fertil Res Pract. (2015) 1:5. doi: 10.1186/2054-7099-1-5 28620510 PMC5415188

[28] Neal SA Morin SJ Franasiak JM Goodman LR Juneau CR Forman EJ . Preimplantation genetic testing for aneuploidy is cost-effective, shortens treatment time, and reduces the risk of failed embryo transfer and clinical miscarriage. Fertil Steril. (2018) 110:896–904. doi: 10.1016/j.fertnstert.2018.06.021 30316435

[29] Eliner Y Foley B Bayer SR Thornton KL Penzias AS Sakkas D . The impact of preimplantation genetic testing for aneuploidy on time to live birth in *in vitro* fertilization. Fertility Sterility. (2025) 124:281–9. doi: 10.1016/j.fertnstert.2025.04.006 40222699

[30] Melo P Dhillon-Smith R Islam M Devall A Coomarasamy A . Genetic causes of sporadic and recurrent miscarriage. Fertil Steril. (2023) 120:940–4. doi: 10.1016/j.fertnstert.2023.08.952 37648143

[31] Suzumori N Sugiura-Ogasawara M . Genetic factors as a cause of miscarriage. Curr Med Chem. (2010) 17:3431–7. doi: 10.2174/092986710793176302 20712563

[32] Vialard F Boitrelle F Molina-Gomes D Selva J . Predisposition to aneuploidy in the oocyte. Cytogenet Genome Res. (2011) 133:127–35. doi: 10.1159/000324231 21335956

[33] Deng J Hong H Zhao Q Nadgauda A Ashrafian S Behr B . Preimplantation genetic testing for aneuploidy in poor ovarian responders with four or fewer oocytes retrieved. J Assist Reprod Genet. (2020) 37:1147–54. doi: 10.1007/s10815-020-01765-y 32285297 PMC7244670

[34] Somigliana E Busnelli A Paffoni A Vigano P Riccaboni A Rubio C . Cost-effectiveness of preimplantation genetic testing for aneuploidies. Fertil Steril. (2019) 111:1169–76. doi: 10.1016/j.fertnstert.2019.01.025 30777289

