# Supplementary material for: PGT-A versus expectant management for fertile patients with recurrent pregnancy loss and prior aneuploidy: a dual-center, real-world study of 5-year cumulative pregnancy outcomes
Source: Front Endocrinol (Lausanne). 2026 Aug 27;17:1926944. doi: 10.3389/fendo.2026.1926944 (PMC13558092; doi:10.3389/fendo.2026.1926944)

Supplementary Figure 1

Variable	N	OR (95% CI)	P_value	P for interaction
Overall	216	0.27 (0.13, 0.53)	<0.001	
Maternal age				0.127
< 35 years	145	0.38 (0.17, 0.87)	0.022	
≥ 35 years	71	0.12 (0.03, 0.52)	0.004	
Parity				0.984
0	174	0.27 (0.12, 0.60)	0.001	
1	42	0.26 (0.05, 1.44)	0.125	
Number of prior miscarriages				0.047
2	114	0.12 (0.04, 0.41)	<0.001	
≥ 3	102	0.55 (0.21, 1.41)	0.212	
Ovarian reserve				0.073
FSH < 7 IU/L	161	0.38 (0.16, 0.87)	0.023	
FSH ≥ 7 IU/L	55	0.09 (0.02, 0.35)	<0.001	

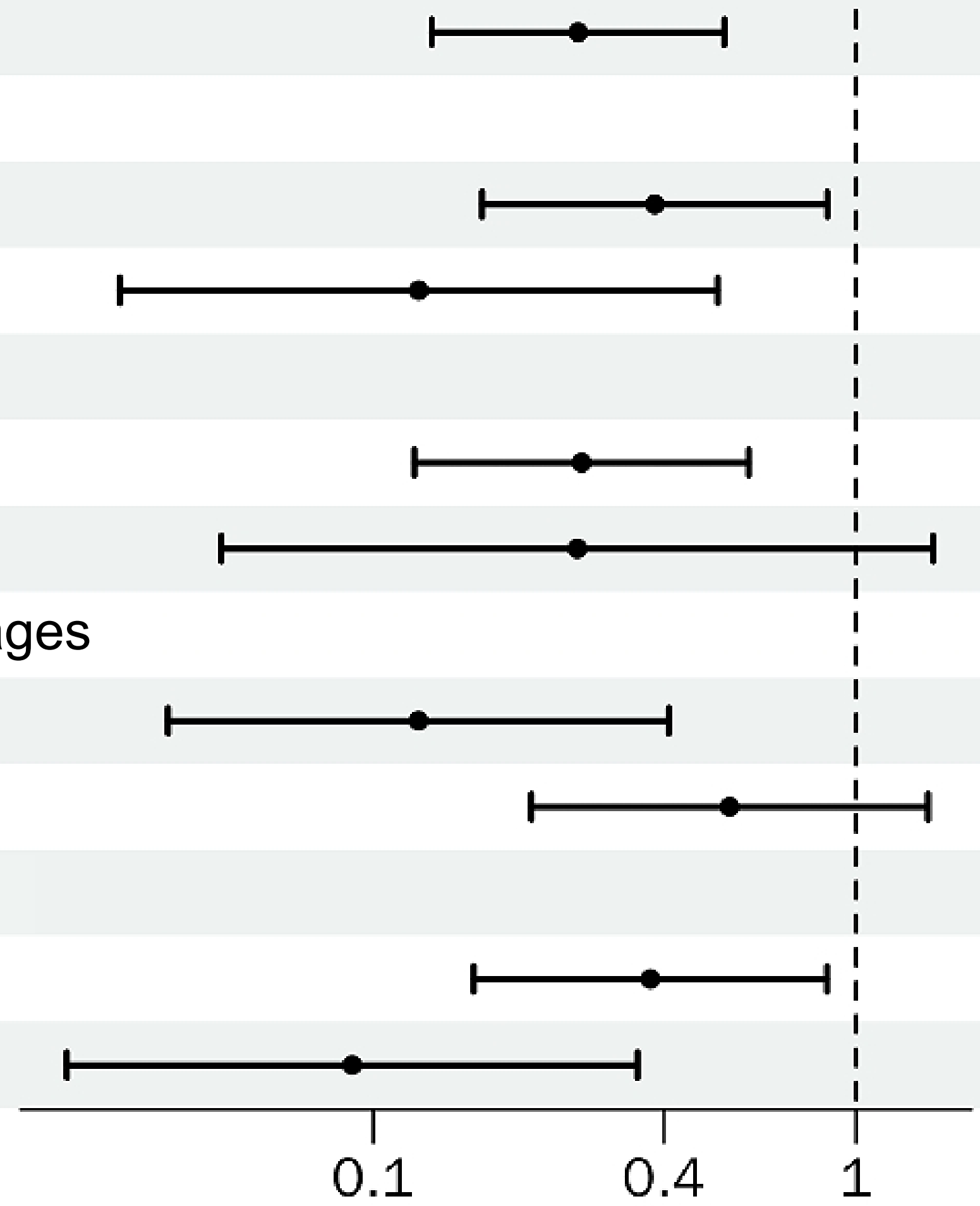

Supplement: Supplementary file 1 [file DataSheet1.pdf]
