# Supplementary material for: PGT-A versus expectant management for fertile patients with recurrent pregnancy loss and prior aneuploidy: a dual-center, real-world study of 5-year cumulative pregnancy outcomes
Source: Front Endocrinol (Lausanne). 2026 Aug 27;17:1926944. doi: 10.3389/fendo.2026.1926944 (PMC13558092; doi:10.3389/fendo.2026.1926944)
Supplement: Supplementary file 2 [file DataSheet2.pdf]

Supplementary Figure 2

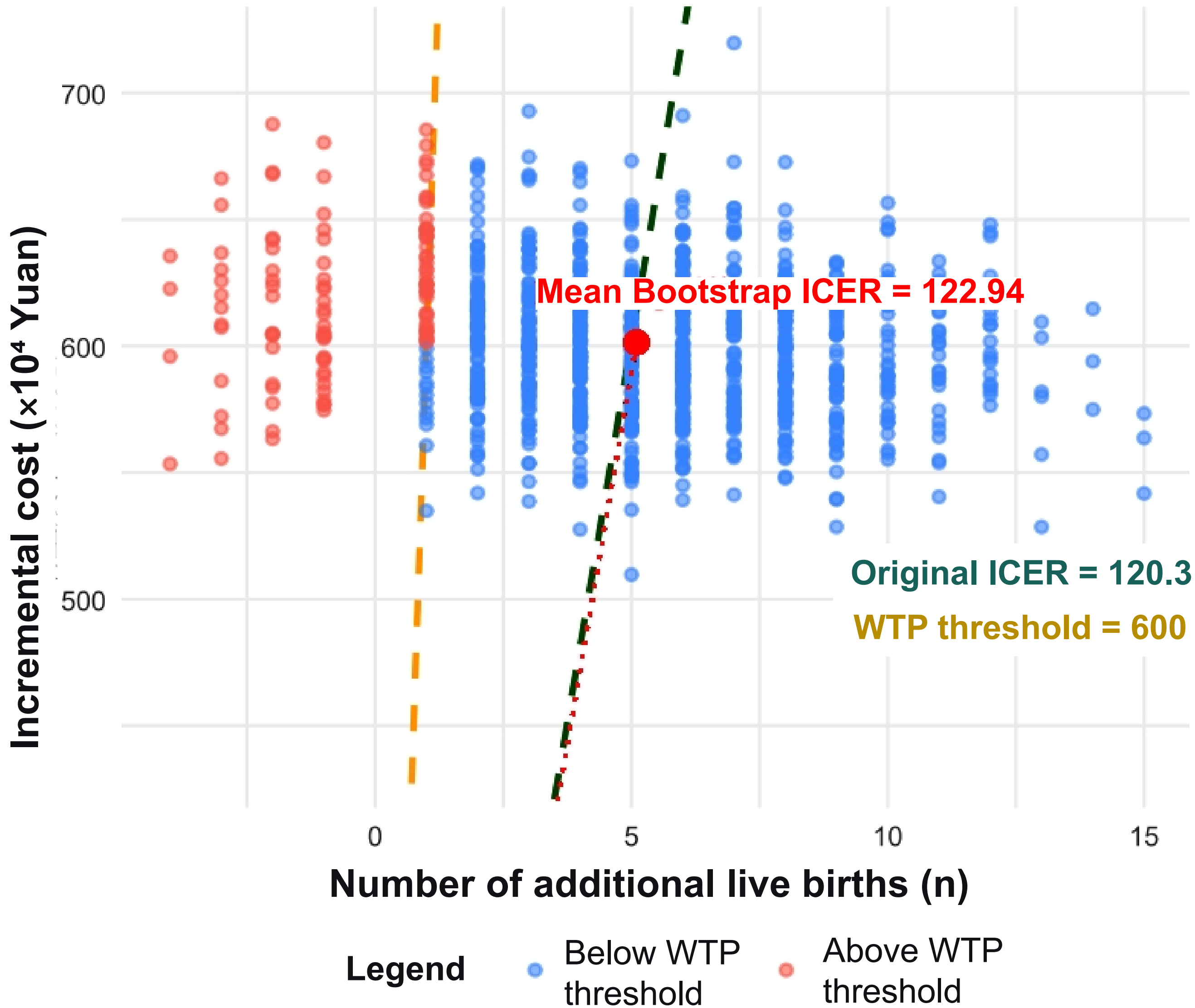

Cost-effectiveness plane for each additional live birth（CEP）
