# Supplementary material for: PGT-A versus expectant management for fertile patients with recurrent pregnancy loss and prior aneuploidy: a dual-center, real-world study of 5-year cumulative pregnancy outcomes
Source: Front Endocrinol (Lausanne). 2026 Aug 27;17:1926944. doi: 10.3389/fendo.2026.1926944 (PMC13558092; doi:10.3389/fendo.2026.1926944)
Supplement: Supplementary file 4 [file Table1.docx]

**Supplementary Table 1.** PGT‑A cycle and embryo biopsy outcomes

| **Parameter** | **Value** |
| --- | --- |
| Patients initiating PGT‑A, n | 145 |
| Total oocyte retrieval cycles, n | 231 |
| Patients with ≥2 retrievals, n (%) | 58 (40.0%) |
| Total oocytes retrieved, n | 2887 |
| Oocytes per patient, mean ± SD (95% CI) | 19.9 ± 10.9 (18.1–21.7) |
| Blastocysts per patient, mean ± SD (95% CI) | 6.8 ± 4.2 (6.1–7.5) |
| Blastocysts biopsied, n | 968 |
| Euploid, n (%) | 516 (53.3%) |
| Aneuploid, n (%) | 290 (30.0%) |
| Segmental abnormal, n (%) | 81 (8.4%) |
| Mosaic, n (%) | 67 (6.9%) |
| Inconclusive, n (%) | 14 (1.4%) |
| Euploid blastocysts per patient, mean ± SD (95% CI) | 3.6 ± 2.5 (3.2–4.0) |
| Patients with no euploid embryo, n (%) | 13 (9.0%) |
| Frozen‑thawed single embryo transfers, n | 191 |
| Transfers per patient, mean (range) | 1.3 (0–4) |

**Supplementary Table 2.** Stratified outcomes by maternal age (intention‑to‑treat population)

|  | **Age < 35 years** | | | **Age ≥ 35 years** | | |
| --- | --- | --- | --- | --- | --- | --- |
|  | **PGT** | **EM** | ***P*** | **PGT** | **EM** | ***P*** |
| N | 93 | 51 |  | 52 | 19 |  |
| CLLB within 12 months | 57 (61.3%) | 42 (80.8%) | 0.016 | 19 (36.5%) | 16 (84.2%) | <0.001 |
| CLLB within 24 months | 74 (79.6%) | 44 (84.6%) | 0.45 | 36 (69.2%) | 16 (84.2%) | 0.21 |
| CLLB within 36 months | 82 (88.2%) | 46 (88.5%) | 0.96 | 40 (76.9%) | 16 (84.2%) | 0.51 |
| CLB up to 5 years | 85 (91.4%) | 46 (88.5%) | 0.77 | 42 (80.0%) | 16 (84.2%) | 0.74 |
| TPLB, months | 8.0 (5.1, 14.0 ) | 3.0 (1.0, 4.6) | <0.001 | 11.1 (5.8, 18.8) | 3.0 (1.7, 5.0) | <0.001 |
| Time to live birth, months | 15.8 (13.1, 25.8) | 11.3 (10.0, 13.6) | <0.001 | 21.2 (14.0, 27.9) | 11.2 (9.8, 13.2) | <0.001 |
| Gestational age at birth, weeks | 37 (37, 39) | 39 (38, 40) | 0.002 | 38 (38, 39) | 39 (38, 40) | 0.14 |
| ITT: intention-to-treat; CLLB: conception leading to live birth; CLB: cumulative live birth; TPLB: time to pregnancy leading to live birth. | | | | | | |

**Supplementary Table 4.** Baseline characteristics of the per‑clinical‑pregnancy cohort (based on actual conception mode)

| **Demographics** | **PGT** | **EM** | ***P*** |
| --- | --- | --- | --- |
| N (per clinical pregnancy) | 122 | 89 |  |
| Female age, y | 33 (31, 36) | 33 (30, 34) | 0.26 |
| Male age, y | 34 (31, 37) | 33 (31, 36) | 0.98 |
| BMI, kg/m^2^ | 21.0 (20.2, 23.0) | 21.1 (20.1, 22.4) | 0.66 |
| Basal FSH , IU/L | 5.8 (4.8, 6.8) | 5.7 (4.9, 7.3) | 0.50 |
| Parity | 19 (15.6%) | 20 (22.5%) | 0.30 |
| The number of prior miscarriages | 3 (2, 3) | 2 (2, 3) |  |
| 2 | 57 (46.7%) | 53 (59.6%) | 0.50 |
| 3 | 44 (36.1%) | 25 (28.1%) |  |
| ≥ 4 | 21 (17.2%) | 11 (12.4%) |  |
| The number of prior aneuploid miscarriage | 1 (1, 1) | 1 (1, 1) |  |
| 1 | 107 (87.7%) | 79 (88.8%) | 0.98 |
| 2 | 15 (12.3%) | 10 (11.2%) |  |
| BMI: body mass index; FSH: follicle‑stimulating hormone. | | | |

**Supplementary Table 5.** Miscarriage outcomes by per‑clinical‑pregnancy and exploratory per‑patient ITT analyses

| **Primary analysis: per clinical pregnancy** | | | | | | |
| --- | --- | --- | --- | --- | --- | --- |
|  | **PGT‑A** | **EM** | **Absolute difference** | **aOR** | **95% CI** | ***P* value** |
| Overall miscarriage (n=211) | 9/122 (7.4%) | 11/89 (12.4%) | –5.0% | 0.42 | 0.15, 1.14 | 0.09 |
| Age <35 years (n=146) | 6/79 (7.6%) | 9/67 (13.4%) | –5.8% | 0.30 | 0.08, 1.09 | 0.06 |
| Age ≥35 years (n=65) | 3/43 (7.0%) | 2/22 (9.1%) | –2.1% | 1.30 | 0.14, 12.57 | 0.79 |
| **Exploratory analysis: per patient ITT (full cohort, n=216)** | | | | | | |
|  | **PGT‑A** | **EM** | **Absolute difference** | **aOR** | **95% CI** | ***P* value** |
| Overall cumulative miscarriage (full cohort，n=216) | 10/145 (6.9%) | 10/71 (14.1%) | –7.2% | 0.32 | 0.11, 0.94 | 0.04 |
| Age <35 years (n=144) | 6.5% (6/93) | 17.6% (9/51) | –11.1% | 0.14 | 0.03, 0.58 | 0.006 |
| Age ≥35 years (n=71) | 7.8% (4/52) | 5.3% (1/19) | + 2.5% | 1.96 | 0.15, 25.3 | 0.60 |
| For the per‑clinical‑pregnancy analysis, multivariate adjustment included age, BMI, parity, prior miscarriage count, prior aneuploid miscarriage count, and basal FSH.  The per‑patient ITT analyses are descriptive and based on the unmatched full cohort (age‑subgroup denominators are the number of patients in each stratum). | | | | | | |

**Supplementary Table 6. Cost-Effectiveness Analysis Results of the PGT-A Group and Expectant Management Group**

| Measure | PGT-A Group | Expectant Management Group |
| --- | --- | --- |
| Mean cost per participant (10,000 CNY) | 9.23 (8.40,10.12) | 0.11 (0.08,0.14) |
|  |  |  |
| Live birth-related outcomes | | |
| Live birth rate (n/N) | 67/71 | 62/71 |
| Cost-effectiveness ratio | 9.83 | 0.13 |
| ICER per additional live birth | 120.38 | — |
| Mean bootstrapped ICER (95% CI) | 122.94 (-579.41,624.51) | — |
|  |  |  |
| Miscarriage-related outcomes | | |
| Early miscarriage rate (n/N) | 4/71 | 10/71 |
| Cost-effectiveness ratio | 10.15 | 0.13 |
| ICER per miscarriage avoided | 150.48 | — |
| Mean bootstrapped ICER (95% CI) | 116.76 (-599.46,622.51) | — |

**Supplementary Table 7.** Sensitivity analysis of cost-effectiveness estimates using different annual discount rates

| Discount Rate | Mean Cost per Patient, PGT-A (CNY) | Mean Cost per Patient, EM (CNY) | Mean Cost Difference per Patient, PGT-A vs EM (CNY) | ICER per Additional Live Birth (10,000 CNY) | ICER per Miscarriage Prevented (10,000 CNY) |
| --- | --- | --- | --- | --- | --- |
| 0% | 92,303.51 | 1,104.60 | 91,198.90 | 120.38 | 150.48 |
| 5% | 87,821.24 | 1,032.73 | 86,788.51 | 114.56 | 143.20 |
| 8% | 85,365.11 | 994.26 | 84,370.85 | 111.37 | 139.21 |
